# Abnormal Serum Bilirubin/Albumin Concentrations in Dementia Patients With Aβ Deposition and the Benefit of Intravenous Albumin Infusion for Alzheimer’s Disease Treatment

**DOI:** 10.3389/fnins.2020.00859

**Published:** 2020-09-03

**Authors:** Xiaomei Zhong, Yuning Liao, Xinru Chen, Naikeng Mai, Cong Ouyang, Ben Chen, Min Zhang, Qi Peng, Wanyuan Liang, Weiru Zhang, Zhangying Wu, Xingxiao Huang, Caijun Li, Hong Chen, Weimin Lao, Chang-E Zhang, Xuejun Wang, Yuping Ning, Jinbao Liu

**Affiliations:** ^1^Department of Geriatric Psychiatry, The Affiliated Brain Hospital of Guangzhou Medical University (Guangzhou Huiai Hospital), Guangzhou, China; ^2^Protein Modification and Degradation Lab, SKLRD, School of Basic Medical Sciences, Affiliated Cancer Hospital of Guangzhou Medical University, Guangzhou, China; ^3^Institute of Neuroscience, The Affiliated Brain Hospital of Guangzhou Medical University (Guangzhou Huiai Hospital), Guangzhou, China; ^4^Guangzhou Yihe Nursing Home, Guangzhou, China; ^5^Guangzhou Songhe Nursing Home, Guangzhou, China; ^6^Division of Basic Biomedical Sciences, Sanford School of Medicine, University of South Dakota, Vermillion, SD, United States

**Keywords:** bilirubin, dementia, Aβ deposition, albumin, Alzheimer’s disease

## Abstract

**Background:**

Our previous study in animal models revealed that bilirubin could induce Aβ formation and deposition. Bilirubin may be important in neurodegenerative dementia with Aβ deposition. Hence, lowering the concentration of the free bilirubin capable of crossing the blood brain–barrier may benefit the treatment of Alzheimer’s disease (AD).

**Objectives:**

The objectives of this study were to examine the change in the serum bilirubin and albumin concentrations of dementia patients with Aβ deposition, and to determine the effects of intravenous administration of albumin in the treatment of AD.

**Methods:**

Bilirubin and albumin concentrations in dementia patients with Aβ deposition were examined. Cell viability and apoptosis were determined in dopaminergic neuron-like cells MN9D treated with bilirubin in the presence of diverse concentrations of serum. Human albumin at a dose of 10 g every 2 weeks for 24 weeks was administered intravenously to AD patients to examine the effect of albumin on AD symptoms.

**Results:**

Significantly higher indirect bilirubin (IBIL) concentrations, lower albumin concentrations, and higher ratio of IBIL to albumin (IBIL/ALB) were observed in dementia patients with Aβ deposition, including AD, dementia with Lewy bodies, and general paresis of insane. *In vitro* assays showed that bilirubin-induced injury in cultured dopaminergic neuron-like cells negatively depends on the concentration of serum in the culture medium. General linear model with repeated measures analysis indicated a main effect of group on the change in albumin concentrations and Alzheimer’s Disease Cooperative Study Activities of Daily Living Inventory scale (ADCS-ADL) scores, and the main effect of time and group, and group-by-time interaction on the change of Clinical Dementia Rating Scale–Sum of Boxes (CDR-SB) scores. Analysis of the combined data of the entire 28 weeks of assessment period using the area under curve convincingly showed significantly improvements in the change of albumin concentrations, ADCS-ADL scores, and CDR-SB scores.

**Conclusion:**

IBIL and the IBIL/ALB ratio are significantly higher in dementia patients with Aβ deposition, and intravenous administration of albumin is beneficial to AD treatment.

**Trial Registration:**

The intervention study was registered at http://www.chictr.org.cn (ChiCTR-IOR-17011539). Date of registration: June 1, 2017.

## Introduction

Around the world, 50 million people are living with dementia, and one new case is expected to develop every 3 s. The number of dementia patients will triple to 152 million in 2050 (Patterson, 2018; World Alzheimer Report, 2018). Neurodegenerative, vascular, and infectious pathologies are proposed to drive the progression to dementia. Neurodegenerative diseases are the major cause of cognitive impairments and can ultimately lead to dementia. Alzheimer’s disease (AD) and dementia with Lewy bodies (DLB) are the most common forms of neurodegenerative dementia, both of which are characterized by misfolded β-amyloid (Aβ) and deposition of Aβ peptide in senile plaques (Aβ deposition) (Glenner et al., 1984; Edison et al., 2008).

Recent research found that metabolic disturbances of liver function were involved in the pathophysiology of AD, showing that the liver function markers were associated with AD-associated A/T/N biomarkers (Nho et al., 2019). However, the causal pathways remain unknown (Nho et al., 2019). Bilirubin is one of the important liver function markers. The toxic effects of bilirubin may contribute to the pathophysiology of AD. Mechanisms associated with the toxic effects of bilirubin include inhibition of enzyme systems and inhibition of cell regulatory reactions (Hansen, 2001). Impairment in the ubiquitin–proteasome system (UPS) was suggested to play a role in neurodegeneration (Bedford et al., 2008). Our previous study has demonstrated that the neurotoxicity of bilirubin is associated with its proteasome inhibition properties. Clinically relevant elevation of bilirubin concentrations can act as an endogenous proteasome inhibitor and inhibit UPS-mediated protein degradation, which may affect the degradation of Aβ and lead to its abnormal accumulation (Hong et al., 2014). In addition, our prior study using primary hippocampal neurons and animal models has shown that bilirubin induces Aβ deposition and formation, and tau hyperphosphorylation, by activating GSK-3β, CDK5, and JNK, increasing the expression of amyloid-β protein precursor (AβPP) γ-secretase PS2, and decreasing the expression of α-secretase ADAM17 (Chen et al., 2020).

Given the vital function of bilirubin in inducing Aβ production, bilirubin may be important in the genesis of neurodegenerative dementia with Aβ deposition. However, it has not been determined whether bilirubin concentrations are abnormal in various types of dementia with Aβ deposition. In patients with AD, related studies have obtained controversial results over the last few decades (Kim et al., 2006; Cankurtaran et al., 2013; Ahmed et al., 2014; Hatanaka et al., 2015; Vasantharekha et al., 2017), and no study has explored the impact of bilirubin concentrations in patients with DLB. General paresis of the insane (GPI), a common type of infectious dementia caused by direct invasion of spirochetes, has proven to be associated with Aβ deposition (Miklossy, 2008). No study has reported the bilirubin concentrations in patients with GPI. The bilirubin concentrations in various types of dementia with Aβ deposition remain to be established.

Over the last 20 years, drug development for the treatment of AD has been focused on altering Aβ aggregate accumulation and the “toxic” actions of these aggregates, destroying soluble Aβ oligomers, and preventing tau accumulation. However, these strategies have failed and have been eventually abandoned (Kozin et al., 2018). Despite the challenges associated with combating the pathological change in symptomatic AD patients, more efforts need to be made to identify new therapeutic approaches. Bilirubin induces Aβ deposition and tau hyperphosphorylation, resulting in AD-like learning and memory injuries in animal models (Chen et al., 2020). We hypothesize that measures to down-regulate bilirubin concentrations in the central nervous system (CNS) may have clinical benefits on improving the cognitive and behavioral functions of AD patients.

Bilirubin entry into the brain is facilitated by low serum albumin concentrations, hyperbilirubinemia, or increased permeability of the blood–brain barrier (BBB) (Hansen, 2001). Almost all serum bilirubin is bound by albumin, and only unconjugated bilirubin is able to pass through the BBB and gain access to the CNS. Up-regulation of peripheral serum albumin may lower the concentrations of unconjugated bilirubin by increasing the bilirubin binding capacity in the serum, which may decrease the rate of bilirubin entry into the brain. Thus, intravenous administration of albumin in AD patients is expected to decrease the peripheral concentrations of unconjugated bilirubin and thereby yield potentially therapeutic benefits to AD patients.

In this study, we first performed a cross-sectional case control study to determine the bilirubin and albumin concentrations in dementia patients with Aβ deposition. Serum bilirubin and albumin concentrations were measured in three types of dementia with Aβ deposition (AD, DLB, and GPI). As bilirubin concentrations are reported to be associated with the risk of silent cerebral infarction (Li et al., 2014) and schizophrenia (Pae et al., 2004; Vitek et al., 2010), we included patients with vascular dementia (VD) and elderly patients with schizophrenia for comparisons. Second, we explored the benefit of serum on decreasing the injury induced by bilirubin in cultured cells. Third, we performed an intervention study to examine the effects of intravenous administration of albumin in AD patients.

## Methods

The Ethics Committee of the Affiliated Brain Hospital of Guangzhou Medical University (Guangzhou Huiai Hospital) approved this study. A written informed consent was obtained from the participants or their nearest relatives. The intervention study was registered at http://www.chictr.org.cn (ChiCTR-IOR-17011539).

### Samples

#### Cross-Sectional Case Control Study

In the cross-sectional case control study, data were drawn from the database of cognitive disorder of the Affiliated Brain Hospital of Guangzhou Medical University (Guangzhou Huiai Hospital). Participants were identified using the following inclusion criteria: (1) diagnosis of AD, DLB, GPI, VD, elderly patients with schizophrenia, or normal elderly controls; (2) availability of complete data of serum concentrations of total bilirubin (TBIL), direct bilirubin (DBIL), and alanine transaminase (ALT); and (3) normal liver function (ALT concentrations were less than or equal to 40 U/L). The diagnosis of AD and DLB was made according to the NINCDS-ADRDA criteria for the clinical diagnosis of probable AD (Mckhann et al., 1984) and the McKeith criteria for the clinical diagnosis of probable DLB (Bonanni et al., 2006), respectively. The diagnoses of VD and schizophrenia were made according to the criteria of the International Classification of Diseases (ICD-10). As there is no gold standard for the clinical diagnosis of GPI, we followed the same criteria used in our previous study (Zhong et al., 2013).

#### Cell Viability and Cell Death Analysis

Cell viability analysis was performed using MTS (CellTiter 96 Aqueous One Solution Reagent, Promega) assay as previously reported (Liao et al., 2019). Briefly, mouse dopaminergic neuron-like cells MN9D (American Type Culture Collection) were seeded on a 96-well plate for 24 h at a concentration of 2000 cells/well. Cells were treated with bilirubin (Sigma-Aldrich, Inc., St. Louis, MO, United States) presented in diverse concentration of fetal bovine serum (FBS) for 48 h. Equal MTS reagent was added in each well in the dark, and cells were incubated for 3 h. The absorbance of optical density was measured with a microplate reader (Sunrise reader, Tecan, Männedorf, Switzerland) at a wavelength of 490 nm from three independent experiments.

Apoptotic analysis was performed using Annexin-V-FITC and propidium iodide (PI) staining assay kit (Keygen Company, Nanjing, China) as previously described (Liao et al., 2018). Briefly, MN9D cells were seeded on a 6-well plate for 24 h and exposed to bilirubin presented in a diverse concentration of FBS for 48 h. Cells were collected and washed three times with cold PBS, and resuspended with the staining mixture at a ratio of a binding buffer (500 μl)/annexin V-FITC reagent (5 μl)/PI (5 μl) in each incubation for 30 min. The stained cells were determined with flow cytometry.

Analysis of protein expression was performed using western blot as previously reported (Liao et al., 2017). Briefly, equal proteins extracted from MN9D cells exposed to bilirubin presented in diverse concentration of FBS for 48 h were separated with the 12% SDS–PAGE and then transferred to polyvinylidene difluoride (PVDF) membranes. Subsequently, the blots were blocked with 5% milk for 1 h. Primary antibodies including antinuclear poly (ADP-ribose) polymerase (PARP, #9532, Cell Signaling Technology, Beverly, MA, United States) and anti-GAPDH (BS60630, Bioworld Technology, Inc., Louis Park, MN, United States) and horseradish peroxidase-conjugated secondary antibodies were used to incubate the blots for 1 h accordingly. The blots were indirectly reacted to the ECL detection reagents and exposed to X-ray films (Kodak, Japan).

#### Intervention Study

In the intervention study, AD patients were recruited from the Guangzhou Songhe Nursing Home and the Guangzhou Yihe Nursing Home. Eligible participants were 50 to 90 years of age, who met the standardized criteria for probable mild or moderate AD (Mckhann et al., 1984), had a score of between 10 and 26 on the Mini-Mental State Examination (MMSE), and had normal liver function (the ALT level was less than or equal to 40 U/L). All the patients received appropriate medical and neurologic evaluations, including magnetic resonance imaging or computed tomography, to exclude patients who had severe or unstable medical disorders, and alternative causes of dementia. None of the patients included in the intervention study received any antidementia drug.

Patients in the treatment group received intravenous human albumin 10 g (50 ml of 20% human albumin) (CSL Behring, United States) every 2 weeks for 24 weeks, with a total of 13 intravenous albumin administrations. The AD patients in the blank control group did not receive any treatment for dementia.

Assessment of cognition was performed with the use of the MMSE (range from 0 to 30) (Folstein et al., 1975) and a 12-item cognitive subscale of the Alzheimer’s Disease Assessment Scale (ADAS-cog; scores range from 0 to 75) (Xia, 2009). Lower scores of MMSE and higher scores of ADAS-cog indicate a worse cognition. Evaluation of daily function was performed with the 23-item version of the Alzheimer’s Disease Cooperative Study Activities of Daily Living Inventory scale (ADCS-ADL; scores range from 0 to 78) (Galasko et al., 1997). In the ADCS-ADL, lower scores indicate a worse function. Assessment of the neuropsychiatric symptoms was performed with the use of the Neuropsychiatric Inventory (NPI; scores range from 1 to 144) (Cummings, 1997). In the NPI, higher scores indicate more severe symptoms. Assessment of dementia severity was performed with the Clinical Dementia Rating Scale–Sum of Boxes (CDR-SB; scores range from 0 to 18) (O’bryant et al., 2008). Higher scores of CDR-SB indicate severe dementia. Cognitive, functional, and neuropsychiatric symptoms and dementia severity measures were evaluated at baseline and at weeks 4, 8, 12, 16, 20, 24, and 28. The efficacy outcomes were the change from baseline in the MMSE, ADAS-cog, NPI, ADCS-ADL, and CDR-SB scores at week 28.

Blood samples were collected at baseline and at weeks 8, 16, and 24. Total serum bilirubin (TBIL), direct bilirubin (DBIL), total protein (TP), and albumin concentrations were measured on a Beckman Coulter AU5800 system (Beckman Coulter, Inc., Brea, CA, United States) using commercially available assays (the kits for TBIL, DBIL, TP, and albumin were from Maccura, China). The values of the indirect bilirubin (IBIL) were calculated by subtracting the DBIL values from the TBIL values.

### Statistical Analysis

All statistical computations were performed using the Statistical Package for Social Sciences (IBM SPSS) 22.0 version (SPSS, Chicago, IL, United States).

In the cross-sectional case control study, non-normally distributed data were log-transformed to ensure a normal distribution. The measurement data among the groups were calculated using a one-way ANOVA followed by an LSD *post hoc* test or Kruskal-Wallis test, followed by a post hoc test. The age, gender, serum ALT concentrations, and serum albumin concentrations were controlled for as covariates in the general linear model (GLM) for the analysis of the bilirubin concentrations. The correlations of the measured values were examined using Pearson’s correlation coefficients.

In the *in vitro* study, data are presented as mean ± SEM from three independent experiments where applicable. To determine statistical probabilities, unpaired Student’s *t*-tests or one-way ANOVA is used where appropriate.

In the intervention study, intention-to-treat (ITT) analyses were performed. Analyses of repeated measures, including the change from baseline of TP, albumin, and bilirubin values, and the change from baseline of the symptom measure scales were conducted by GLM repeated measures, with group as the between-subjects factor and time of assessment as the within-subject factor. The time point difference was examined using an LSD *post hoc* test.

The area under curve (AUC) of each parameter was used for the analysis of the combined data of the entire 24 or 28 weeks of assessment period. The time course data were converted to AUC for each patient, and the differences in AUC among the two groups were evaluated using independent-samples *t*-tests and Mann–Whitney *U*-tests, where appropriate. A *P*-value of less than 0.05 was considered to be statistically significant.

## Results

### Demographic and Clinical Characteristics in the Cross-Sectional Study

In the cross-sectional case control study, a total of 352 participants (94 AD, 29 DLB, 99 GPI, 30 VD, 69 elderly patients with schizophrenia, and 31 normal elderly controls) met all the inclusion criteria. The MMSE scores were available in 205 participants (67 AD, 18 DLB, 78 GPI, 11 VD, and 31 normal elderly controls). The demographic and clinical characteristics are presented in Table 1. The age, education, gender, the MMSE score, and the serum concentrations of ALT, TP, and albumin were significantly different among these six groups (all *P* < 0.001). The age of the GPI group was lower than that of the normal elderly controls, while the age of the VD patients was higher than that of the normal elderly controls. The level of education in the AD and DLB groups was lower than that of the normal elderly control group. The GPI group included significantly more men than the normal elderly control group. Serum ALT concentrations in the elderly schizophrenia group were significantly lower than those of the normal elderly control group.

**TABLE 1:** Patient demographic and clinical characteristics in the cross-sectional study.

	**AD**	**DLB**	**GPI**	**VD**	**SP**	**NC**	***F/Z/*χ*^2^***	***P***
*N*	94	29	99	30	69	31		
Age (years)	70.7 ± 10.1	70.1 ± 9.2	54 ± 9.3***	78.5 ± 7.0**	66.0 ± 8.5	66.1 ± 5.8	39.5	<0.001
Education (years)	7.3 ± 4.6*	6.4 ± 3.7*	8.9 ± 3.8	10.3 ± 4.4	9.8 ± 2.3	10.3 ± 3.6	22.3	<0.001
Gender (female)	57(60.6%)	14(48.3%)	17(17.2%)***	14(46.7%)	47(68.1%)	18(58.1%)	56.1	<0.001
Serum ALT (U/L)	20.1 ± 8.2	21.4 ± 8.2	21.3 ± 8.2	15.2 ± 5.5**	14.9 ± 7.8**	20.4 ± 6.2	8.15	<0.001
Serum TP (g/L)	65.7 ± 6.2***	65.7 ± 5.5***	68.5 ± 6.5***	66.3 ± 7.2***	66.2 ± 5.4***	73.3 ± 7.9	8.15	<0.001
Serum albumin (g/L)	40.0 ± 3.8***	40.4 ± 5.3**	41.2 ± 3.9*	37.3 ± 3.9***	37.6 ± 3.2***	43.0 ± 1.5	14.3	<0.001
MMSE scores	8.0 ± 6.6***	11.2 ± 7.3***	14.6 ± 6.8***	9.6 ± 7.0***	NA	27.2 ± 1.7	51.9	<0.001

Data are presented as mean ± SD. Compared to normal controls, *P < 0.05, **P < 0.01, ***P < 0.001. AD, Alzheimer’s disease; DLB, dementia with Lewy body; GPI, general paresis of insane; VD, vascular dementia; SP, schizophrenia; NC, normal control; ALT, alanine transaminase; ALB, albumin; TP, total protein; MMSE, Mini-Mental State Examination; NA, not applicable. Kruskal-Wallis test was used for the comparisons of the age and education, and one-way ANOVA was used for the comparisons of the concentration of ALT, TP and albumin, and the MMSE scores.

### Serum TP and Albumin Concentrations in the Cross-Sectional Study

Serum TP concentrations in the AD, DLB, GPI, VD, and elderly patients with schizophrenia groups (all *P* < 0.001), as well as the serum albumin concentrations in the AD (*P* < 0.001), DLB (*P* = 0.009), GPI (*P* = 0.019), VD (*P* < 0.001), and elderly patients with schizophrenia (*P* < 0.001) groups were significantly lower than those of the normal elderly control group.

### Serum Bilirubin Concentrations in the Cross-Sectional Study

**FIGURE 1:**
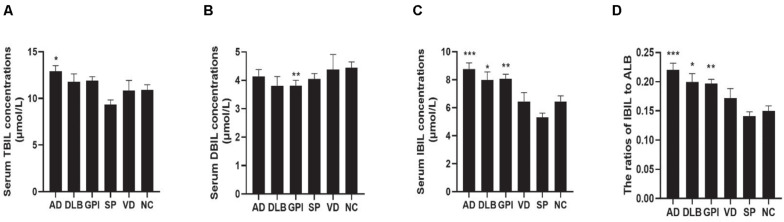
Serum TBIL, DBIL, and IBIL concentrations and the ratio of IBIL/ALB in the cross-sectional study. Serum bilirubin concentrations in AD, DLB, GPI, VD, SP, and NC groups: **(A)** serum TBIL concentrations; **(B)** serum DBIL concentrations; **(C)** serum IBIL concentrations; **(D)** IBIL/ALB ratio. Data presented in the figures are means and SEM. GLM was used for the analysis of the bilirubin concentrations, and the age, gender, serum ALT concentrations, and serum albumin concentrations were controlled for as covariates. Compared to controls, *P < 0.05, **P < 0.01, and ***P < 0.001. AD, Alzheimer’s disease; DLB, dementia with Lewy body; GPI, general paresis of the insane; VD, vascular dementia; SP, schizophrenia; NC, normal control; TBIL, total bilirubin; DBIL, direct bilirubin; IBIL, indirect bilirubin; ALB, albumin.

The serum TBIL concentrations were not different between the AD and the normal elderly control groups in the one-way ANOVA analysis, while after controlling for age, gender, and serum concentrations of ALT and albumin, the TBIL concentrations in the AD group were significantly higher than those in the normal elderly control group (Figure 1A). The serum DBIL concentrations in the GPI group were higher than those of the normal elderly controls (Figure 1B). One-way ANOVA showed that the serum IBIL concentrations and the ratios of IBIL to albumin (IBIL/ALB) were significantly higher in the AD, DLB, and the GPI groups when compared to those of the normal elderly control group. These results were still robust after controlling for age, gender, and serum concentrations of ALT and albumin (Figures 1C,D).

The male AD patients had significantly higher TBIL (*t* = 2.251, *P* = 0.029) and DBIL (*t* = 2.544, *P* = 0.013) concentrations than females. The male elderly schizophrenia patients showed higher DBIL (*t* = 2.096, *P* = 0.04) concentrations than females. No gender differences in TBIL, DBIL, or IBIL concentrations were found in VD or DLB patients.

### Correlations of Serum Bilirubin Concentrations and MMSE Scores in the Cross-Sectional Study

Of the 222 dementia patients with Aβ deposition, the MMSE scores were available in 163 patients (67 AD, 78 GPI, and 18 DLB). The results of the Pearson’s correlation with the 163 samples showed that the serum TBIL concentrations (*r* = −0.179, *P* = 0.022), serum IBIL concentrations (*r* = −0.170, *P* = 0.03), and the IBIL/ALB ratios (*r* = −0.191, *P* = 0.014) were inversely correlated to the MMSE scores.

### *In vitro* Study of Diverse Serum Concentrations on Bilirubin-Induced Cell Death

**FIGURE 2:**
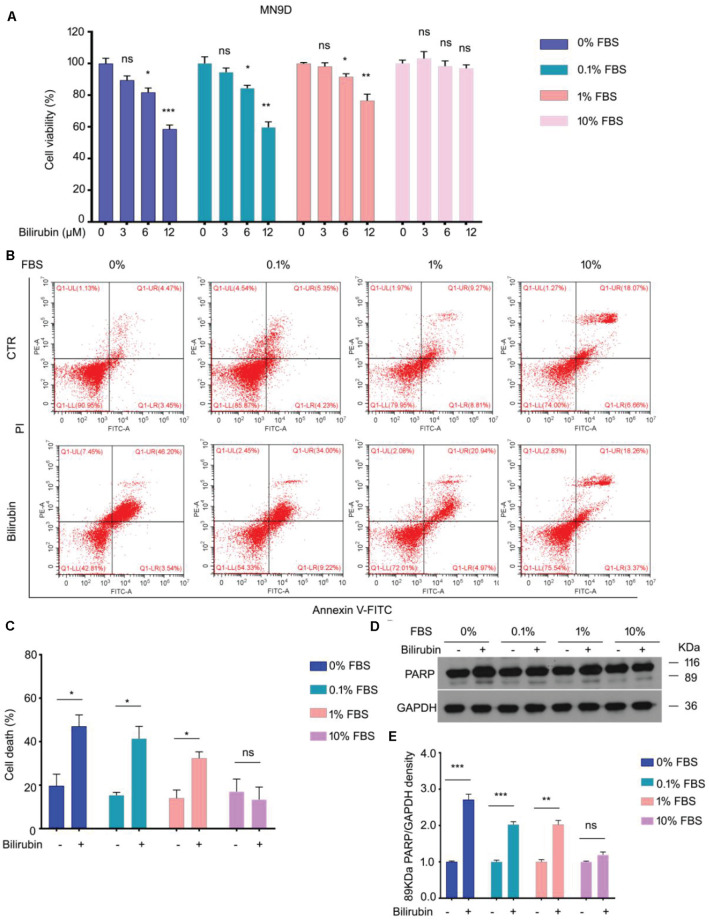
The effect of serum concentrations in the culture medium on bilirubin-induced cell death. **(A)** Cell viability assays of MN9D cells treated with bilirubin that presented in increasing concentrations of fetal bovine serum (FBS) for 48 h. Data shown in the figures are means and SEM (*n* = 3). Unpaired Student’s *t*-tests and one-way ANOVA were used. Compared to controls, *P < 0.05, **P < 0.01, and ***P < 0.001; ns, not significant. **(B)** Apoptotic assays of MN9D cells treated with bilirubin (12 μM) that presented in increasing concentrations of FBS for 48 h. Quantitative data shown in **(C)** are means and SEM (*n* = 3). Unpaired Student’s *t*-tests and one-way ANOVA were used. Compared to controls, *P < 0.05; ns, not significant. **(D)** Immunoblot assay of PARP in MN9D cells treated with bilirubin (12 μM) that presented in increasing concentrations of FBS for 48 h. GAPDH, loading control. Quantitative data shown in **(E)** are means and SEM (*n* = 3). Unpaired Student’s *t*-tests and one-way ANOVA were used. Compared to controls, **P < 0.01, ***P < 0.001, ns, not significant.

In the *in vitro* study, MTS assay was performed to determine the cell viability of MN9D cells treated with bilirubin in the presence of a medium with 0, 0.1, 1, or 10% FBS for 48 h. The results show that bilirubin significantly decreased the viability of MN9D cells in the presence of a medium with 0, 0.1, 1% FBS, but not 10% FBS (Figure 2A). Our flow cytometry and western blot assays revealed that cell death induction by bilirubin was weaken with the increase in the FBS concentration (Figures 2B–E). These *in vitro* findings demonstrate that bilirubin-induced injury in dopaminergic neuron-like cells negatively depends on the concentration of serum.

### Demographic and Clinical Characteristics in the Intervention Study

**FIGURE 3:**
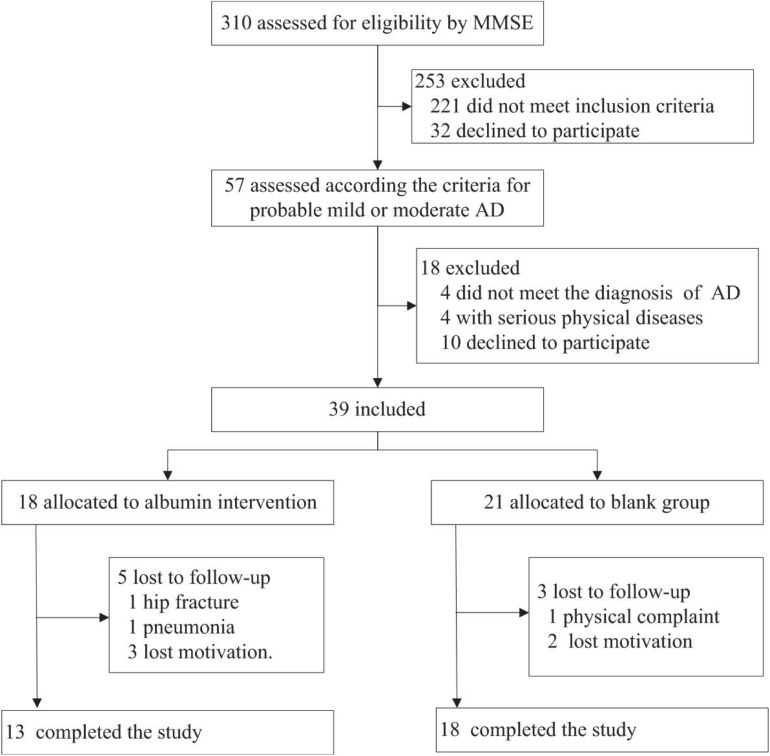
Patient flow diagram. Flow diagram for the progress of patients through the trial.

In the intervention study, 310 elderly subjects in the nursing home were screened, and a total of 39 AD patients were included; 18 received intravenous albumin and 21 were assigned to the blank control group (Figure 3). In the blank control group, the ADAS-cog score in 1 patient at baseline was lacking, and 1 patient at weeks 8 and 2 patients at week 20 dropped out of the study. In the intervention group, the serum albumin value in 1 patient at baseline was lacking, and 2 patients at week 12, 1 patient at week 16, and 2 patients at week 28 dropped out of the study. Thirteen patients in the treatment group and 18 patients in the control group completed the study. The demographic and clinical characteristics of the two groups at baseline are presented in Table 2. There were no statistically significant differences in age, gender, education, serum concentrations of TBIL, DBIL, IBIL, TP, and albumin, and the scores of MMSE, ADAS-cog, NPI, ADCS-ADL, and CDR-SB between the intervention and the blank control groups (all *P* > 0.05) (Table 2).

**TABLE 2:** Baseline demographic and clinical characteristics in the intervention study.

	**Treatment group**	**Control group**	***t/z***	***P***
*N*	18	21		
Age (years)	81.8 ± 4.4	82.7 ± 5.5	−0.5	0.6
Education (years)	5.3 ± 4.2	6.5 ± 5.9	−0.7	0.5
Gender (female)	13(72.2%)	18(85.7%)	1.0	0.4
Serum TBIL (μmol/L)	10.5 ± 3.7	11.0 ± 4.0	−0.4	0.6
Serum DBIL (μmol/L)	4.2 ± 1.6	3.9 ± 1.4	−0.5	0.5
Serum IBIL (μmol/L)	6.2 ± 2.5	7.0 ± 3.2	−0.2	0.7
IBIL/ALB	0.167 ± 0.061	0.203 ± 0.125	−0.1	0.8
Serum TP (g/L)	72.8 ± 6.1	73.0 ± 4.2	−0.1	0.8
Serum ALB (g/L)	36.2 ± 5.0	38.1 ± 8.2	−0.8	0.4
MMSE scores	17.3 ± 5.0	18.1 ± 4.9	−0.2	0.7
ADAS-cog scores	24.9 ± 11.6	22.5 ± 10.8	0.6	0.5
ADCS-ADL scores	47.1 ± 20.0	56.2 ± 14.0	−1.6	0.1
NPI scores	10.5 ± 14.8	6.1 ± 8.8	−1.1	0.2
CDR-SB scores	6.1 ± 4.0	3.7 ± 2.8	−1.5	0.1

*Data are presented as mean ± SD. TBIL, total bilirubin; DBIL, direct bilirubin; IBIL, indirect bilirubin; TP, total protein; ALB, albumin; MMSE, Mini-Mental State Examination; ADAS-cog, Alzheimer’s Disease Assessment Scale—Cognitive Subscale; ADCS-ADL, Alzheimer’s Disease Co-operative Study—Activities of Daily Living; NPI, Neuropsychiatric Inventory; CDR-SB, Clinical Dementia Rating Scale—Sum of Boxes.*

### Serum TP, Albumin, and Bilirubin Concentrations in the Intervention Study

**FIGURE 4:**
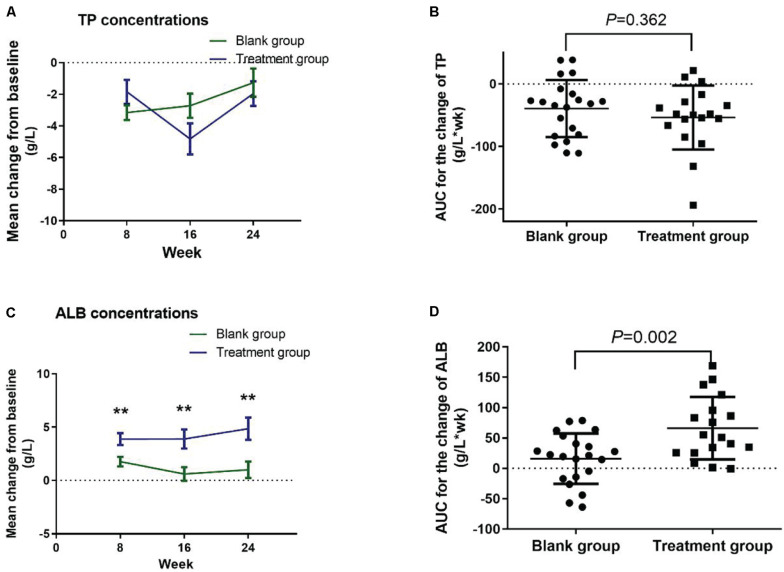
Change in the serum TP and albumin concentrations from baseline in the intervention study. **(A)** Change in serum TP concentrations; **(B)** AUC for the change of T*P* concentr*a*tions; **(C)** change in serum albumin concentrations; **(D)** AUC for the change of albumin concentrations. Data were analyzed using GLM repeated measures, with an LSD *post hoc* test. **P < 0.01 versus blank group. Data presented in the figures are means and SEM. TP, total protein; ALB, albumin; AUC, area und*e*r curve.

There were no significant group differences in the TP concentrations, as well as the AUC (Figures 4A,B), between the treatment and blank control groups. GLM repeated measures revealed a main effect of group on the change in albumin concentrations (*F* = 11.596, *P* = 0.002), while no effect of time or group-by-time interaction was found. LSD *post hoc* analyses indicated that the change of albumin concentration scores at weeks 8, 16, and 24 was significantly higher in the treatment group compared with the blank control group (*P* = 0.006, *P* = 0.004, *P* = 0.005, respectively; Figure 4C). The AUC of change in albumin concentrations significantly increased in the treatment group when compared with the blank control group (*t* = 3.316, *P* = 0.002; Figure 4D).

**FIGURE 5:**
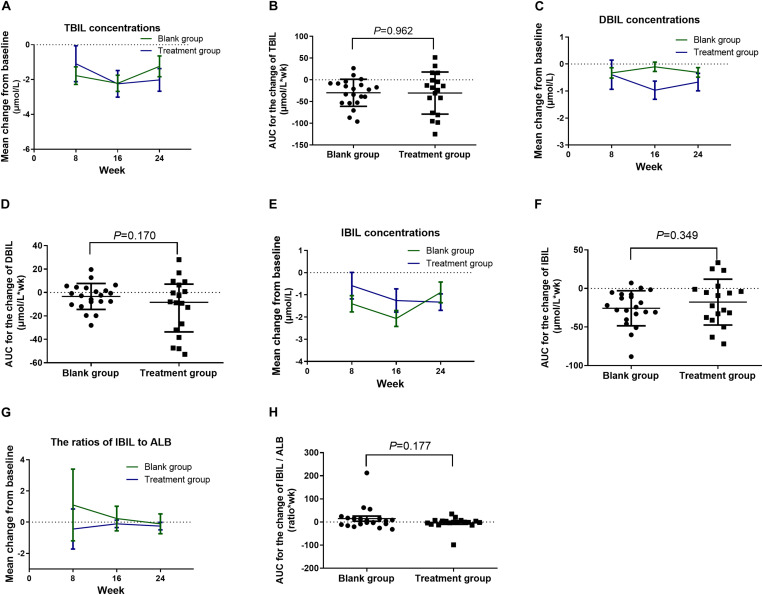
Change in the serum TBIL, DBIL, and IBIL concentrations and the IBIL/ALB ratio from baseline in the intervention study. **(A)** Change in serum TBIL concentrations; **(B)** AUC for the change of TBIL concentrations; **(C)** change in serum DBIL concentrations; **(D)** AUC for the change of DBIL concentrations; **(E)** change in serum IBIL concentrations; **(F)** AUC for the change of IBIL concentrations; **(G)** change in IBIL/ALB ratios; **(H)** AUC for the change of IBIL/ALB. The time course data **(A,C,E,G)** were converted to AUC for each patient **(B,D,F,H).** Data were analyzed using GLM repeated measures, with an LSD *post hoc* test. Data presented in the figures are means and SEM or median with range. TBIL, total bilirubin; DBIL, direct bilirubin; IBIL, indirect bilirubin; ALB, albumin; AUC, area under curve.

No significant differences were noted in the TBIL, DBI, IBIL, and IBIL/ALB, as well as the respective AUC between the treatment and blank control groups (Figures 5A–H).

### Change in the MMSE, ADAS-cog, NPI, ADCS-ADL, and CER-SB Scores From Baseline in the Intervention Study

**FIGURE 6:**
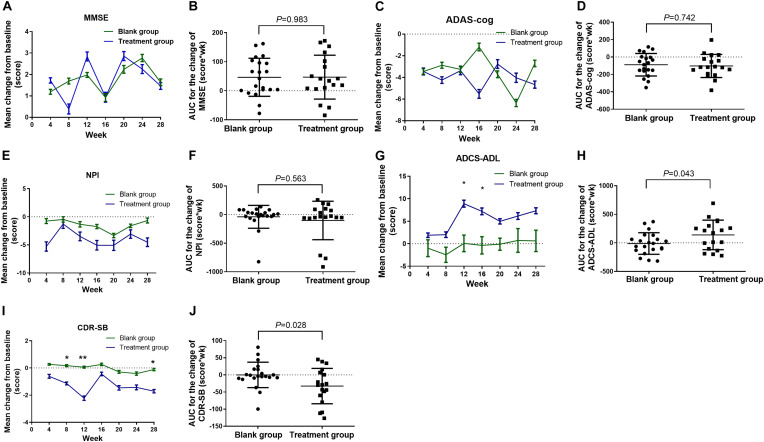
Change in the MMSE, ADAS-cog, NPI, ADSC-ADL, and CDR-SB scores from baseline in the intervention study. **(A)** Change in the MMSE scores; **(B)** AUC for the change of MMSE scores; **(C)** change in the ADAS-cog scores; **(D)** AUC for the change of ADAS-cog scores; **(E)** change in the NPI scores; **(F)** AUC for the change of NPI scores; **(G)** change in the ADCS-ADL scores; **(H)** AUC for the change of ADCS-ADL scores; **(I)** change in the CDR-SB scores; **(J)** AUC for the change of CDR-SB scores. The time course data **(A,C,E,G,I)** were converted to area under curve (AUC) for each patient **(B,D,F,H,I)**. Data were analyzed using GLM repeated measures, with an LSD *post hoc* test. *P < 0.05 versus blank group, and **P < 0.01 versus blank group. Data presented in the figures are means and SEM or median with range. MMSE, Mini-Mental State Examination; ADAS-cog, Alzheimer’s Disease Assessment Scale—Cognitive Subscale; NPI, Neuropsychiatric Inventory; ADCS-ADL, Alzheimer’s Disease Co-operative Study—Activities of Daily Living; CDR-SB, Clinical Dementia Rating Scale—Sum of Boxes; AUC, area under curve.

No effect of group or time or group-by-time interaction on the change of MMSE, and no significant difference in the AUC were observed (Figures 6A,B). GLM repeated measures showed a significant group-by-time interaction on the change of ADAS-cog scores (*F* = 3.497, *P* = 0.009), while no main effect of group (*F* = 0.154, *P* = 0.697) or time (*F* = 0.719, *P* = 0.637) was observed. No significant difference was found among every time point on the change of ADAS-cog scores (Figure 6C) and the AUC (Figure 6D) in the treatment group when compared with the blank control group. No effect of group or time or group-by-time interaction on the change of NPI, and no significant difference in the AUC were observed (Figures 6E,F). GLM repeated measures revealed a main effect of group on the change in ADCS-ADL scores (*F* = 4.353, *P* = 0.044), while no effect of time or group-by-time interaction was found. LSD *post hoc* analyses indicated that the change of ADCS-ADL scores at weeks 12 and 16 was significantly higher in the treatment group compared with the blank control group (*P* = 0.020, *P* = 0.043, respectively; Figure 6G). The AUC of change in ADCS-ADL scores significantly increased in the treatment group when compared with the blank control group (*t* = 2.091, *P* = 0.043; Figure 6H). A significant main effect of time (*F* = 2.846, *P* = 0.025) and group (*F* = 5.405, *P* = 0.026) and group-by-time interaction on the change of CDR-SB (*F* = 2.750, *P* = 0.029) was observed. LSD *post hoc* analyses indicated that the change of CDR-SB scores at weeks 8, 12, and 28 were significantly lower in the treatment group compared with the blank control group (*P* = 0.027, *P* = 0.003, *P* = 0.024, respectively; Figure 6I). The AUC of change in CDR-SB scores significantly lower in the treatment group when compared with the blank control group (*t* = −2.285, *P* = 0.028; Figure 6J).

## Discussion

Given the role of bilirubin in inducing Aβ production and its neurotoxicity shown in experimental studies, bilirubin may be important in neurodegenerative dementia with Aβ deposition. To our knowledge, this is the first study to examine serum bilirubin concentrations in multiple forms of dementia with Aβ deposition, and more importantly, this represents the first preliminary study to examine the benefits of intravenous albumin infusion for the treatment of AD. We found that serum IBIL values and the IBIL/ALB were significantly higher in the two most common forms of neurodegenerative dementia (AD and DLB), as well as infectious dementia [GPI, which has been proven to be associated with Aβ deposits (Miklossy, 2008)]. Intravenous administration of albumin is beneficial to the improvement of daily function and attenuates the severity of the dementia in AD patients.

An increased accumulation of misfolded Aβ has been shown in AD, DLB, and GPI (Glenner et al., 1984; Edison et al., 2008; Miklossy, 2008), while the pathogenesis of VD and elderly schizophrenia patients is not characterized by increased Aβ deposits in the brain (Arnold et al., 1994; Purohit et al., 1998; Roman, 2002). Accordingly, we found higher IBIL concentrations and higher IBIL/ALB in patients with AD, DLB, and GPI, but not in VD or elderly schizophrenia patients. In contrast to our work, some studies described lower concentrations of total bilirubin in AD patients compared to healthy subjects (Kim et al., 2006; Hatanaka et al., 2015; Vasantharekha et al., 2017), while other studies reported no differences (Cankurtaran et al., 2013; Ahmed et al., 2014; Nho et al., 2019). Among these studies, only the study by Vasantharekha et al. (2017) explored the concentrations of DBIL and IBIL and showed decreased IBIL concentrations in AD patients. Only IBIL is able to cross the BBB freely and to yield an effect on the brain. Hence, IBIL values and the IBIL/ALB may be more suitable for the determination of the effect of circulatory bilirubin on the brain. Our results in AD patients are in line with previous findings by Kimpara and colleagues, showing that cerebrospinal fluid (CSF) bilirubin concentrations in AD patients increased significantly when compared with controls (Kimpara et al., 2000). The correlations of serum bilirubin concentrations with MMSE scores in dementia patients with Aβ deposition revealed by our study indicate that the IBIL concentrations and the ratios of IBIL/ALB may have a negative impact on the cognitive function.

Our previous study demonstrated that clinically relevant elevated concentrations of bilirubin can inhibit UPS-mediated protein degradation (Huang et al., 2017). Malfunctioning of the UPS in AD may affect the degradation of Aβ and lead to an abnormal accumulation of Aβ (Hong et al., 2014). In addition, our study in primary hippocampal neurons and in animal models showed that bilirubin works as an endogenous pathological factor for AD, inducing Aβ production and tau hyperphosphorylation (Chen et al., 2020). Our cross-sectional case-controlled study indicates that increased IBIL concentrations and a greater IBIL/ALB ratio are particularly segregated with the dementia with Aβ deposition patients. These results, in combination with our findings in primary hippocampal neurons and in animal models (Chen et al., 2020), suggest that increased serum concentrations of IBIL may play an important role in the etiology of dementia with Aβ deposition.

Our *in vitro* investigations unravel that bilirubin significantly inhibits cell viability and triggers cell death in mouse dopaminergic neuron-like cells cultured in medium with low serum but fails to induce these effects in the medium with 10% FBS. A rational explanation is that the damage effects were caused by the unconjugated bilirubin, and these effects were decreased with the elevation of serum. These findings, consistent with the previous study (Huang et al., 2017) and our clinical observations in AD patients, indicate that increase of serum albumin may alleviate the neurotoxicity induced by unconjugated bilirubin that enters into the brain in AD. Therefore, increasing the concentration of serum albumin to decrease the unconjugated bilirubin concentrations in the circulation could be a strategy for confronting the bilirubin-induced neurotoxicity and AD-like injuries.

In the intervention study, we found significant change in the albumin concentrations after intravenous infusion of human albumin at a dose of 10 g every 2 weeks. Higher albumin concentration reflects a higher capacity of serum albumin to bind with bilirubin and thereby keeps bilirubin in serum, which may result in decreased levels of bilirubin entry into the brain. Change of ADCS-ADL and CDR-SB scores was consistently observed. Analysis of the combined data of the entire 28 weeks of assessment period using the AUC of each parameter as the index convincingly shows significant improvements in the change of ADCS-ADL and CDR-SB scores in the treatment group compared with the blank control group. These results suggest that intravenous infusion of human albumin benefits on daily function and dementia severity in patients with mild to moderate AD. However, intravenous administration of human albumin at a dose of 10 g every 2 weeks in patients with mild or moderate AD did not significantly reduce the cognitive decline as measured by the MMSE and ADAS-cog at 28 weeks. There may be several reasons for these results: (1) The 28-week period of this intervention study may not be long enough for albumin to affect the cognition of AD by binding to bilirubin. It may require longer time for intravenous human albumin to slow down the development of cognitive impairment. (2) The pathogenic cascade of AD, starting with accumulation of the Aβ1–42 peptide into oligomeric and fibrillar assemblies, is thought to begin at least one to two decades prior to the cognitive impairment (Langbaum et al., 2013), and the brain has usually undergone extensive degeneration (Golde et al., 2018). Decreased concentrations of bilirubin entering the brain may have limited effects on preexisting Aβ pathology and the cognitive impairment. The optimal time for more successful albumin therapy may be more than 10 years before the appearance of the clinical symptoms of AD (Langbaum et al., 2013; Golde et al., 2018).

The serum bilirubin remained comparable between the treatment and blank control groups after albumin administration. However, we found an assertive evidence that bilirubin-induced neurotoxicity in dopaminergic neuron-like cells was decreased by increasing the concentration of serum, and intravenous administration of human albumin to AD patients benefited on their daily function and dementia severity. We did not observe a direct correlation between the change of serum bilirubin concentrations and the change of symptoms. This may be because we measured the bilirubin concentrations in blood samples but not the CSF samples. We do not know the accurate change of bilirubin concentrations in the brains of AD patients before and after albumin infusion. Further study exploring the association among albumin infusion, change of CSF bilirubin concentrations, and change of AD symptoms is needed.

There are several limitations to the present study. First, the intervention study was not a random controlled trial. Second, the sample size was small. Third, individuals were enrolled with a clinical diagnosis of AD, without CSF-based biomarkers or Aβ imaging to document the pathology of Aβ. Last, the factors affecting BBB opening were not included since the BBB is gradually open with aging. In addition, the participants included in the intervention study suffered from mild or moderate AD, while presymptomatic patients were not included. Future studies involving double-blind, placebo-controlled prevention trials in asymptomatic individuals with documented AD biomarkers are needed.

## Conclusion

Our study found that serum IBIL concentrations and the IBIL/ALB were significantly higher in patients with AD, DLB, or GPI, but not in cases of VD or in elderly schizophrenia patients. These results suggest that the probability for unconjugated bilirubin that can freely cross the BBB under certain conditions such as hypoxia and infection to gain access to the CNS is significantly higher in dementia patients with Aβ deposition. Our results provide further evidence to support our previous finding in primary hippocampal neurons and in animal models, showing that bilirubin could induce Aβ deposition and formation (Chen et al., 2020). Consistent with these experimental and clinical findings, our intervention study shows that intravenous supplementation of human albumin at a dose of 10 g every 2 weeks for 24 weeks yields benefit on daily function and dementia severity in AD patients. Given the unmet medical need for the millions of individuals who will become symptomatic for AD, it is mandatory to identify new therapeutic approaches. To optimize the chances of success, future studies of IBIL/ABL therapy should be initiated in the presymptomatic stage when bilirubin-based neurotoxicity is likely reversible.

## Data Availability Statement

The raw data supporting the conclusions of this article will be made available by the authors, without undue reservation, to any qualified researcher.

## Ethics Statement

The studies involving human participants were reviewed and approved by Affiliated Brain Hospital of Guangzhou Medical University. The patients/participants or their nearest relatives provided their written informed consent to participate in this study.

## Author Contributions

YN and JL designed the study. XZ designed the study, analyzed and interpreted the data, and wrote the manuscript. YL completed the cytological experiment and wrote the manuscript. XC analyzed the data and assessed the subjects. NM, CO, BC, MZ, QP, WLi, WZ, and ZW assessed the subjects. XW participated in data analysis and manuscript revision. C-EZ revised the manuscript. XH, CL, HC, and WLa contributed patients. All authors read and approved the final manuscript.

## Conflict of Interest

The authors declare that the research was conducted in the absence of any commercial or financial relationships that could be construed as a potential conflict of interest.
